# Distinct microbiota assembly and functional patterns in disease-resistant and susceptible varieties of tobacco

**DOI:** 10.3389/fmicb.2024.1361883

**Published:** 2024-03-01

**Authors:** Luhua Yang, Yuan Guo, Hui Yang, Shun Li, Yunzeng Zhang, Cheng Gao, Tian Wei, Likai Hao

**Affiliations:** ^1^Key Laboratory of Marine Environmental Corrosion and Biofouling, Institute of Oceanology, Chinese Academy of Sciences, Qingdao, China; ^2^Key Laboratory of Urban Environment and Health, Ningbo Urban Environment Observation and Research Station, Institute of Urban Environment, Chinese Academy of Sciences, Xiamen, China; ^3^State Key Laboratory of Environmental Geochemistry, Institute of Geochemistry, Chinese Academy of Sciences, Guiyang, China; ^4^Guizhou Academy of Tobacco Science, Guiyang, China; ^5^University of Chinese Academy of Sciences, Beijing, China; ^6^Zhejiang Key Laboratory of Urban Environmental Processes and Pollution Control, CAS Haixi Industrial Technology Innovation Center in Beilun, Ningbo, China; ^7^College of Bioscience and Biotechnology, Yangzhou University, Yangzhou, China; ^8^State Key Laboratory of Microbial Resources, Institute of Microbiology, Chinese Academy of Sciences, Beijing, China; ^9^Bei Bu Zhan Qu CDC, Shenyang, China; ^10^CAS Center for Excellence in Quaternary Science and Global Change, Xi’an, China

**Keywords:** plant microbiota, tobacco microbiome, disease resistance, positive selection, biocontrol

## Abstract

The plant microbiota is believed to be an accessory genome that extends plant functions, forming holobionts together with the host plant. Plant disease resistance, therefore, is inextricably linked with plant microbiota, which play important roles in plant growth and health. To explore the relationship between plant microbiota and disease resistance, we investigated the tobacco microbiome of two varieties with contrasting disease-resistance levels to bacterial wilt and black shank diseases. Comparative microbiome analysis indicated that the resistant variety assembled a distinct microbiota with higher network complexity and diversity. While *Pseudomonas* and *Ensifer,* which contain biocontrol and beneficial members, were enriched in the rhizosphere of the resistant variety, *Ralstonia*, a genus including the known causative pathogen, was enriched in the susceptible variety. Metagenome sequencing revealed that biocontrol functions, such as hydrogen cyanide synthase, pyochelin biosynthesis, and arthrofactin-type cyclic lipopeptide synthetase, were more abundant in the resistant variety. Further analysis indicated that contigs encoding the corresponding genes were mostly assigned to *Pseudomonas*. Among all the metagenome-assembled genomes, positive selection was suggested in the genome assigned to *Pseudomonas* only in the rhizosphere of the resistant variety. The search of biosynthetic gene clusters in the *Pseudomonas* genome revealed a non-ribosomal peptide synthetase, the compound of which was brabantamide A, with known antimicrobial activity. Collectively, our study suggests that the plant microbiota might be involved in microbe-mediated disease resistance. Particularly, our results highlight *Pseudomonas* in the rhizosphere of the disease-resistant variety as a promising biocontrol candidate. Our study may facilitate further screening of bacterial isolates and the targeted design of microbial communities.

## Introduction

Recent studies have highlighted that plants cannot be viewed as standalone entities (Vandenkoornhuyse et al., 2015; Wei and Jousset, 2017) but rather as a part of the functional unit interdependent with their associated microbiota, which is commonly referred to as the “plant holobiont” (Sanchez-Canizares et al., 2017; Trivedi et al., 2020; Lyu et al., 2021). The plant microbiota is believed to extend plant functions as an accessory genome and play critical roles in plant growth and health (Chialva et al., 2022). Consequently, plant microbiota are inextricably linked to plant disease resistance (Vannier et al., 2019), with growing evidence indicating that they can confer disease resistance to host plants at both individual and community levels (Carrion et al., 2019; Choi et al., 2020; Gu et al., 2020; Matsumoto et al., 2021; Li et al., 2022).

Recently, studies have pinpointed the specific plant genes within barley and tomato that actively shape the plant microbiome (Escudero-Martinez et al., 2022; Oyserman et al., 2022), which could mediate disease resistance. This discovery suggests a profound interconnection between the inherent plant disease resistance and reciprocal traits exhibited by rhizobacteria. Therefore, gaining insights into the plant microbiome of varieties with varying disease resistance becomes pivotal for unraveling resistance mechanisms, particularly in plants lacking known host-resistance genes. While most previous studies emphasized the state with the presence of pathogens (Gao et al., 2021; Yin et al., 2021; Gu et al., 2022), very few have evaluated the healthy states under natural conditions. Accordingly, the interplay between the natural microbiome and the resulting disease performance remains elusive.

Tobacco (*Nicotiana tabacum*) is one of the most economically significant agricultural crops worldwide. Bacterial wilt (caused by *Ralstonia solanacearum*) and black shank (caused by *Phytophthora nicotianae*) are two major diseases affecting tobacco plants (Gallup et al., 2018; Ahmed et al., 2022). To explore the relationship between disease resistance and plant microbiota, we carefully selected two tobacco varieties with contrasting disease-resistance levels. We hypothesize that the disease-resistant variety hosts a more diverse and stable natural microbiome, enriched with a higher abundance of beneficial microorganisms. In contrast, the disease-susceptible variety exhibits a less diverse microbiome. These findings aim to contribute to a comprehensive understanding of plant health, potentially enhancing agricultural sustainability.

## Materials and methods

### Sample collection and DNA extraction

Two varieties of flue-cured tobacco (*Nicotiana tabacum*) were chosen for investigation: HD and GZ36. HD, a conventional variety widely cultivated, exhibits high susceptibility to black shank (caused by *Phytophthora nicotianae*) and bacterial wilt (caused by *Ralstonia solanacearum*). In contrast, GZ36, a recently developed variety, showcases remarkable resistance to prevalent tobacco diseases.

The experimental cultivation took place in fields within the tobacco-planting farmlands of the typical karst region in Tianma, Anshun, Guizhou, China (26° 24′ 19.548” N, 106°15′24.588″ E). Destructive sampling was performed on 26 July 2020, precisely during the topping stage, approximately 10 days subsequent to the elimination of terminal buds. The selection of this sampling point was underpinned by two principal considerations. First, the topping stage signifies a pivotal juncture in the growth and production of tobacco plants, marking the transition from the reproductive phase to the vegetative phase. Second, disease resistance typically experiences a decline after the topping stage.

The bulk soil, roots with soil attached, and leaves were sampled and promptly transferred to the laboratory on ice. Triplicates were taken for each variety. The soil cores (at 0–20 cm depth) from each field were collected and pooled as one bulk soil replication, containing approximately 80 g. The plants were further divided into four compartments, as described below. First, the loosely attached soil was removed by shaking. The roots were then placed into a 50-ml Falcon tube with PBS buffer and were washed on a shaking platform for 20 min at 180 rpm. The washing buffer was then subjected to centrifugation (1,500 g, 20 min), and the pellet obtained was defined as the rhizosphere compartment. Roots were transferred to a new 50-ml Falcon tube. After a second washing step (20 min, 180 rpm) with PBS buffer and surface sterilization using 75% ethanol (5 min, 180 rpm), roots were flash-frozen with liquid nitrogen and were defined as the root compartment (root endosphere). The leaves were divided into leaf epiphytes and leaf endophytes in the same way as the roots. All compartments were flash-frozen and were stored at −80 °C. The DNA was extracted using the Mag-MK Soil Genome DNA extraction kit (Sangon, China). In total, 30 samples, including two varieties and five compartments, were obtained.

### Amplicon sequencing

The primer pairs, 799F and 1193R (Chelius and Triplett, 2001) were used for the bacterial 16S rRNA gene amplification to avoid the co-amplification of the chloroplast. The PCR reaction systems contained 25 μL of 2x Premix Taq (Takara Biotechnology, Japan), 1 μL of each primer (10 μM), and 3 μL of DNA (20 ng/μl) template in a volume of 50 μL. The thermocycling program is as follows: 5 min at 94°C for initialization; 30 cycles of 30-s denaturation at 94°C, 30 s annealing at 52°C, and 30-s extension at 72°C, followed by a 10-min final elongation at 72°C. The sequencing libraries were generated using the NEBNext^®^ Ultra^™^ II DNA Library Prep Kit for Illumina^®^ (New England Biolabs, United States) following the manufacturer’s instructions. The quality was assessed on the Qubit@ 2.0 Fluorometer (Thermo Fisher Scientific, United States). In the end, the library was sequenced on an Illumina Nova6000 platform (Illumina, United States).

### Metagenomic sequencing

After quantifying genomic concentrations using the Qubit^™^ dsDNA HS Assay Kit (Thermo Fisher Scientific, United States), libraries with an insert length of approximately 500 bp were prepared with a total of approximately 500 ng of DNA. The DNA of each sample was mechanically sheared to approximately 500 bp fragments using Covaris S220 (Covaris, United States). The sheared DNA was processed using the NEBNext Ultra DNA Library Prep Kit for Illumina (New England Biolabs, United States) for end repair and adaptor ligation. The fragmented DNA was recovered using 1x Hieff NGS^™^ DNA Selection Beads (Yeasen Biotechnology, China). The purified PCR products were assayed using a Qubit 4 Fluorometer (Thermo Fisher Scientific, United States), were pooled with equal concentrations, and were sequenced on the Illumina HiSeq platform (Illumina, United States).

### Bioinformatics

The amplicon sequencing analysis was performed using USEARCH (v11.0.667). After the removal of primers and PhiX contamination, the reads were merged and filtered. The clean reads were denoised using the UNOISE3 algorithm (Edgar, 2016a). The taxonomy assignment of the 16S rRNA gene was performed using the RDP training set (v18) (Cole et al., 2014) with the SINTAX taxonomy prediction algorithm (Edgar, 2016b). The reads assigned to chloroplasts and mitochondria were filtered out.

For metagenome sequencing, the quality control and the removal of plant-derived reads were performed by KneadData (v0.10.0).^1^ The taxonomy was assigned using MetaPhlAn (v3.0.13) (Beghini et al., 2021). The metagenomic reads were assembled by megahit (v1.2.9) (Li et al., 2015). The reads assembled for eukaryotes, viruses, and archaea were filtered out using MMseqs2 (v13.45) (Mirdita et al., 2021). Gene annotation, clustering, and quantification were performed using Prodigal (v1.14.6) (Hyatt et al., 2010), MMseqs2 (v13.45) (Mirdita et al., 2021), and CoverM (v0.3.2),^2^ respectively. Functional annotation of the reassembled bins was conducted using eggNOG-Mapper (v2.0.1) (Huerta-Cepas et al., 2017, 2019).

The genome reconstruction, including binning and refinement, was conducted using MetaWRAP (v1.3.2) (Uritskiy et al., 2018). The taxonomy of bins was checked by GTDK-Tk (v2.1.1) (Chaumeil et al., 2022) with the database release207_v2 (Parks et al., 2022). Bins with a completeness of more than 80% and contamination of less than 5% were considered to be of high quality and were kept for further analysis. The prediction of biosynthetic gene clusters in the metagenome-assembled genomes was performed using antiSMASH (v6.1.1) (Blin et al., 2021) with the default settings.

The metagenomic reads of each bin were recruited using anvi’o v7.1 (Eren et al., 2021) and the described workflow (Shaiber et al., 2020). The variants in metagenomic read recruitment results were calculated using the microbial population genetics framework implemented in anvi’o. The polymorphism rates of individual codon sites from allele frequencies were calculated to infer the synonymous (pS^(site)^) and non-synonymous (pN^(site)^) changes as defined previously (Shenhav and Zeevi, 2020; Kiefl et al., 2022). The ratio of non-synonymous substitution (dN) rates and synonymous substitution (dS) rates (dN/dS) is widely used for studying the selection, which could quantify substitution rates between diverged species (Kryazhimskiy and Plotkin, 2008). In this study, we calculated the per-site rates of synonymous (pS) and non-synonymous polymorphism (pN), as pS and pN could resolve shorter evolutionary timescales than the typical fixation rate. Moreover, pS and pN can be calculated using metagenomic data without complete haplotypes and could specify rates on a per-sample basis, allowing for inter-sample comparisons (Kiefl et al., 2022). In the calculation of pN/pS, the minimum length of contigs was set at 200 bp. The minimum coverage was tenfold, and the minimum occurrence in samples was specified as two. While pN/pS >1 indicates positive selection owing to increased rates of non-synonymous substitutions, pN/pS <1 indicates purifying selection, and pN/pS =1 implies neutral evolution (Rocha et al., 2006).

### Statistics

In the study of alpha diversity, we utilized Hill numbers as the diversity index and used the R package *“hilldiv”* (Alberdi and Gilbert, 2019) for the calculation. Hill numbers, also called effective numbers, were proposed as a unified diversity concept using the scaling parameter q, also known as the diversity order (Chao et al., 2014). The parameter q determines the weight given to the relative abundance of an operational taxonomic unit (OTU) in a community. For instance, when q is 0, the relative abundance is not considered; when q is 1, the OTUs/amplicon sequence variants (ASVs) are weighted precisely according to their relative abundance; and when q is 2, more weight is given to OTUs/ASVs with high relative abundance. The R package *“phyloseq”* was used for ordination studies (McMurdie and Holmes, 2013). The community dissimilarity in PCoA was tested using adonis in the R package *“vegan”* (Oksanen et al., 2013).

The rhizosphere and root endosphere samples were combined as root compartments to construct the molecular ecological networks. Only OTUs with more than 20 reads present in no less than half of the samples were included for correlation calculation. The Spearman correlations were performed using the R package *“psych”* (Revelle, 2019). The cutoff threshold of the correlation and the *p*-value were set at 0.7 and 0.001, respectively. The same approach was adopted for the phyllosphere samples. In total, four co-occurrence networks, each representing one variety (GZ36/HD) of one compartment (root/leaf), were constructed. The nodes were divided into four subcategories according to the values of within-module connectivity (Zi) and among-module connectivity (Pi): (i) highly linked connector nodes (Zi ≤ 2.5, Pi >0.62), (ii) module hubs (Zi > 2.5, Pi ≤0.62), (iii) network hubs (Zi > 2.5, Pi >0.62), and (iv) peripheral nodes (Zi ≤ 2.5, Pi ≤0.62) (Olesen et al., 2007). The nodes that belonged to connectors, module hubs, or network hubs were considered as keystone OTUs. We then calculated cohesion (Herren and McMahon, 2017) as a property of network complexity using the ‘taxa shuffle’ null model. Negative cohesion indicates disparate niches and/or negative interactions between taxa, whereas positive cohesion reflects niche overlap and/or positive interactions (Hernandez et al., 2021; Yuan et al., 2021).

The taxa specifically inhabiting different microhabitats were identified using the linear discriminant analysis effect size (LEfSe) method (Segata et al., 2011) using the visualization web server ImageGP (Chen et al., 2022). The cutoff of the Wilcoxon and Kruskal–Wallis (KW) tests were both set at 0.01. The cutoff LAD value was set at 3. Differences in functional potentials in the two varieties were determined using the R package *“DESeq2”* (Love et al., 2014). The results with an adjusted *p*-value less than 0.05 and a log_2_ fold change of more than two were considered significant.

## Results

### Variety effects were strong in the root microbiota but were less prominent in the phyllosphere

**Figure 1:**
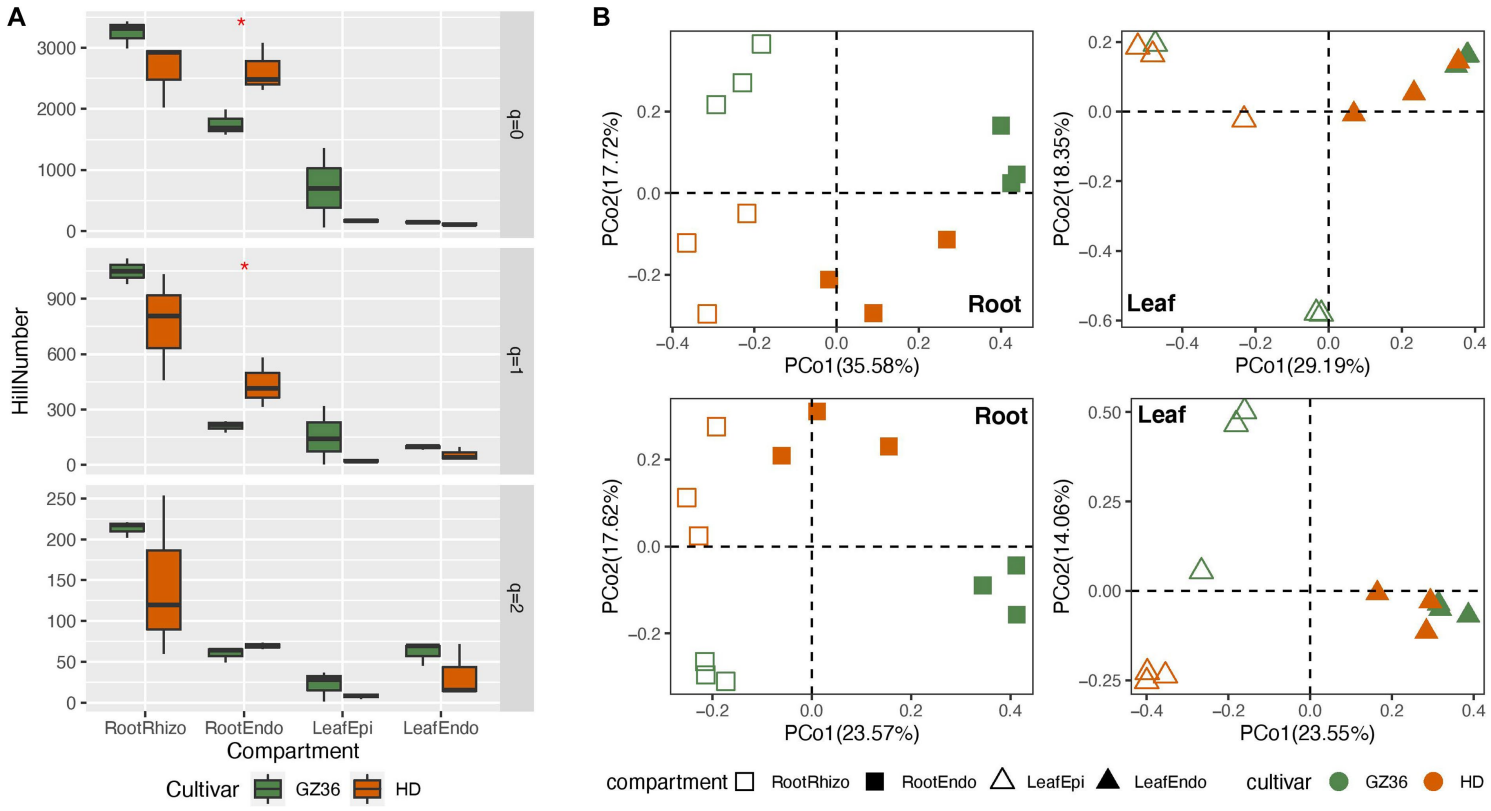
A comparative study of the tobacco microbiome between two varieties with contrasting disease resistance levels. The disease-resistant variety GZ36 was represented by the green color, while the disease-susceptible variety HD was represented by the red color. **(A)** The alpha diversity and **(B)** beta diversity of the root and leaf microbiome associated with the disease-resistant variety GZ36 and the disease-susceptible variety HD. The asterisk symbol in panel **(A)** indicates statistical significance. The different symbols in panel **(B)** represent plant compartments.

In this study, we investigated the root and leaf microbiota of the disease-resistant tobacco variety GZ36 and the disease-susceptible variety HD. In the root samples, contrasting trends of alpha diversity were observed in the rhizosphere and endosphere (Figure 1A). While the diversity of the rhizosphere was higher in GZ36, the diversity of the endosphere was higher in HD. However, statistical differences were only found in the endosphere at *q* = 0 (equivalent to richness) and *q* = 1 (equivalent to Shannon index), despite the strong trends observed. In the phyllosphere, both the epiphytic and endophytic compartments exhibited higher diversity in GZ36, although the results were not statistically significant.

The community structure was further compared using principal coordinate analysis (PCoA) (Figure 1B). Varieties appeared to be the strong drivers of root microbiota, which were corroborated by the permutational multivariate analysis (*adonis*, *p* < 0.01). However, no statistical significance was observed in the phyllosphere microbiota between the two varieties.

**Figure 2:**
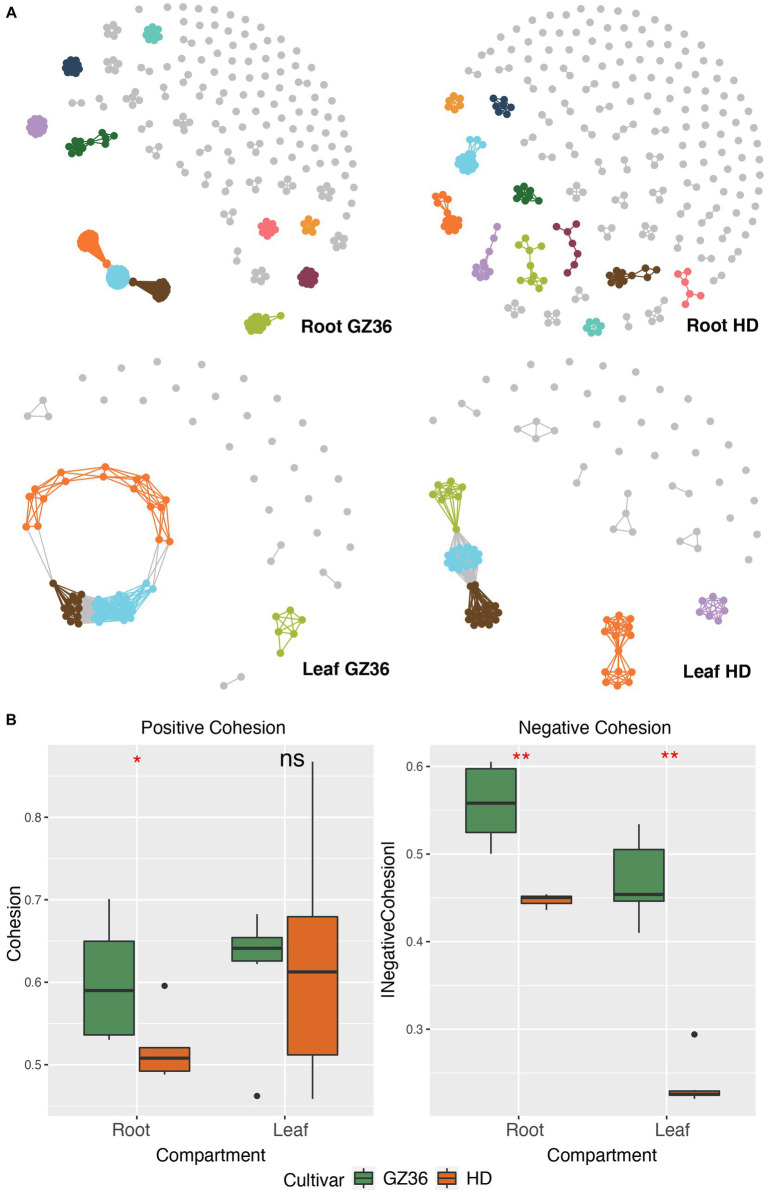
Microbial co-occurrence network of the disease-resistant tobacco variety GZ36 exhibited higher complexity than that of the disease-susceptible variety HD. **(A)** The co-occurrence networks of root and leaf microbiota associated with the two tobacco varieties. **(B)** The corresponding complexity of each co-occurrence network is quantified by the cohesion index. The absolute value of the cohesion index indicates the degree of complexity. The asterisk denotes statistical significance, while ns represents no statistical significance.

Molecular ecological networks were constructed to investigate the microbial patterns of the two varieties in each compartment (Figure 2A). The complexity, i.e., the connectivities of the microbial communities, was quantified using the index of cohesion. The absolute value of negative cohesion was found to be higher in the disease-resistant variety GZ36 in both root and leaf compartments. The positive cohesion, however, only differed in the root compartment (higher in GZ36), whereas no statistical differences were observed in the phyllosphere (Figure 2B).

The keystone OTUs of each network were identified according to their topological features (Supplementary material S1; Supplementary Figure S1). In the root compartment, 30 OTUs were detected as key stone OTUs in GZ36 (Actinobacteria, Bacteroidetes, and Proteobacteria), while 40 OTUs were detected as key OTUs in HD (Acidobacteria, Actinobacteria, Candidatus_Saccharibacteria, Gemmatimonadetes, and Proteobacteria) (Table 1). Although the keystone OTUs appeared more diverse in HD, the phylum Bacteroidetes was found exclusively in GZ36 (Table 1). In the phyllosphere, 29 keystone OTUs were observed in GZ36 (including 3 unassigned, 2 Actinobacteria, 15 Bacteroidetes, 2 Firmicutes, and 7 Proteobacteria), whereas only 8 OTUs (2 unassigned, 2 Bacteroidetes, 2 Firmicutes, and 2 Proteobacteria) were found in HD. A much higher proportion of Bacteroidetes were revealed in the resistant variety GZ36.

**Table 1:** Topological features of the molecular ecological networks, including the number and the taxonomic distribution of key stone OTUs.

Network	Keystone OTUs	Number of Keystone OTUs in each phylum								
		Actinobacteria	Bacteroidetes	Proteobacteria	Firmicutes	Acidobacteria	Candidatus_Saccharibacteria	Gemmatimonadetes	Spirochaetes	unassigned
RootGZ36	30	10	1	17	–	–	–	-	-	2
RootHD	40	17	–	18	–	1	1	1	-	3
LeafGZ36	29	2	15	6	2	–	–	-	1	3
LeafHD	8	–	2	2	2	–	–	-	-	*2*

### Distinct microbiota and KEGG orthologs were enriched in the two tobacco varieties

**Figure 3:**
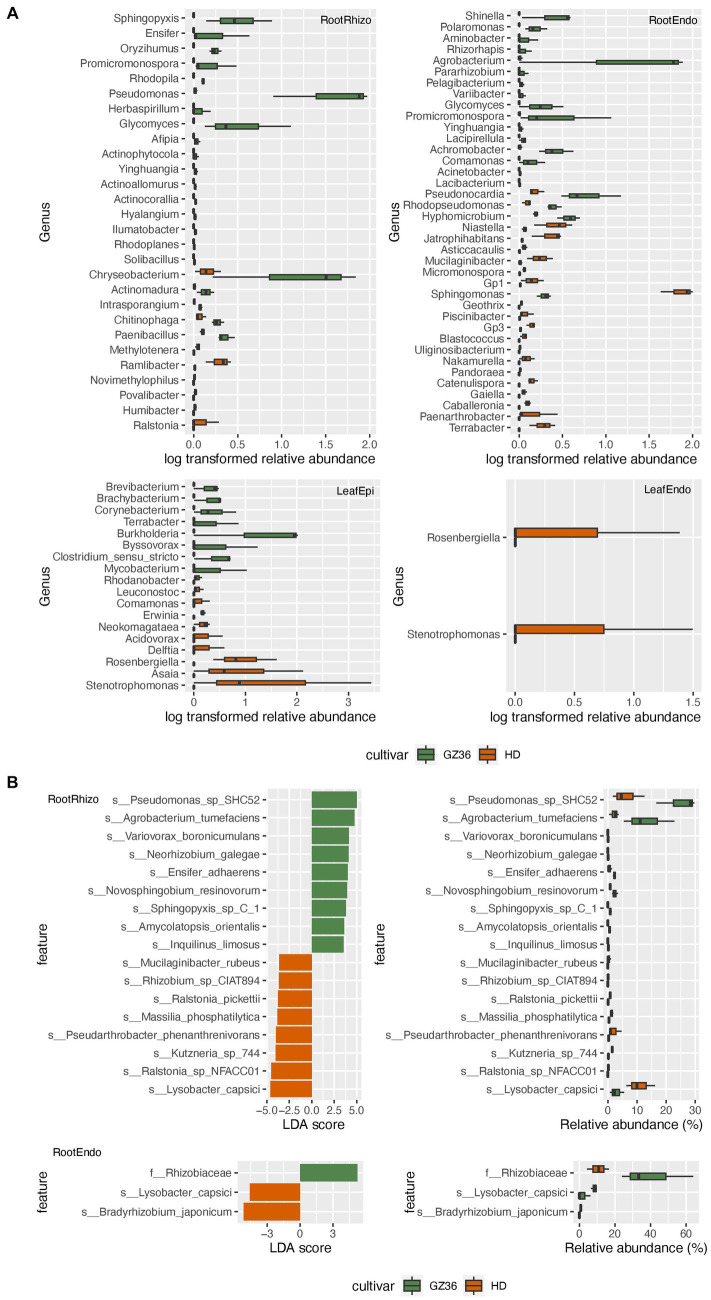
The taxa enriched in each tobacco variety. **(A)** The genera with statistically different abundances between the two varieties based on amplicon sequencing. **(B)** The taxa with statistically different abundances between the two varieties based on metagenome sequencing. The green color denotes the disease-resistant variety GZ36, whereas the red color denotes the disease-susceptible variety HD.

Based on the results of 16S rRNA gene sequencing, 22 genera were found to be more abundant in the rhizosphere of GZ36, among which *Sphingopyxis*, *Ensifer*, *Pseudomonas*, *Glycomyces*, and *Chryseobacterium* were the dominant groups (Figure 3A). In contrast, nine genera displayed higher abundance in the rhizosphere of HD, including *Ralstonia*, *Humibacter*, *Povalibacter*, *Novimethylophilus*, *Ramlibacter*, and *Methylotenera*. In the root endosphere, equal numbers of genera (11 genera) were enriched in each variety. Notably, *Agrobacterium* and *Sphingomonas* appeared prominent in the enriched groups of the two varieties. However, in the phyllosphere, contrasting trends were observed. The genera, namely *Stenotrophomonas* and *Rosenbergiella*, in the endosphere were enriched exclusively in the variety HD. In the epiphytic leaf compartment, 8 genera were more abundantly present in GZ36, while 10 genera showed higher abundance in HD.

A taxonomy comparison based on metagenomic sequencing was also carried out. However, differences between the two varieties were only observed in the root samples but not in the leaf samples (Figure 3B). Similar to the results based on amplicon sequencing, *Pseudomonas*, *Ensifer*, and *Sphingopyxis* were enriched in the rhizosphere of GZ36, whereas two species of *Ralstonia* (*Ralstonia_pickettii* and *Ralstonia_sp_NFACC01*) were enriched in HD.

We further combined the epiphytic and endophytic compartments to form root samples and leaf samples. While similar results were obtained in the root samples as in the rhizosphere, *Stenotrophomonas* and *Kosakonia* were found to be enriched in the phyllosphere of HD (Supplementary material S1; Supplementary Figure S2).

**Figure 4:**
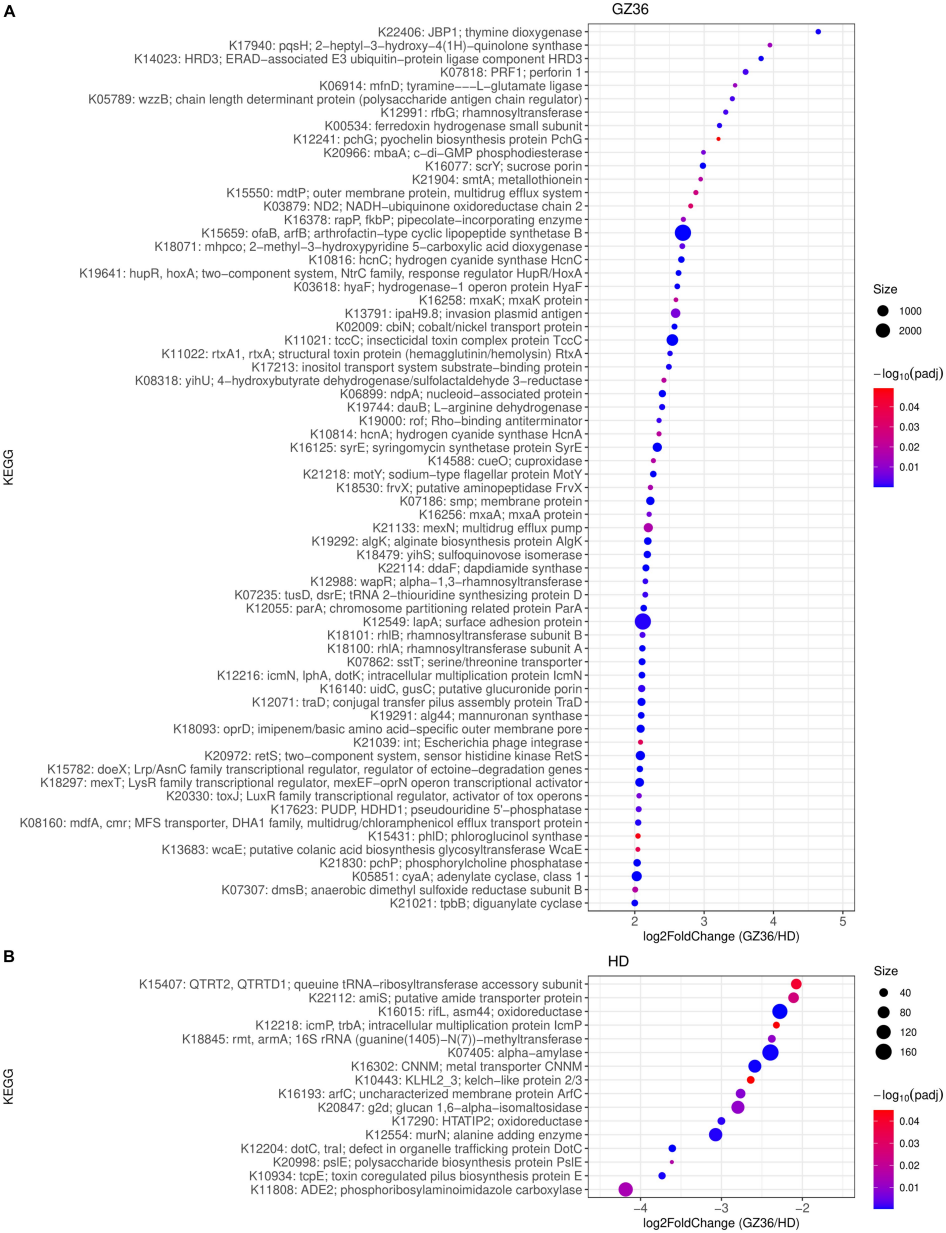
KEGG orthologs (KOs) enriched in **(A)** the disease-resistant variety GZ36 and **(B)** the disease-susceptible variety HD. The size of the dots was in correspondence with the absolute reads number of the KO in the enriched variety. The color of the KOs corresponds to the negative value of the base 10 logarihm of the adjusted *p*-value.

We further investigated the functional differences in root and leaf microbiota between the two varieties. In the rhizosphere, 66 KEGG Orthologs (KOs) showed higher abundance in GZ36, whereas 16 KOs were more abundant in HD (Figure 4). Among the KOs enriched in the rhizosphere of GZ36, two KOs were found to be related to hydrogen cyanide synthase (K10814 hydrogen cyanide synthase HcnA and K10816 hydrogen cyanide synthase HcnC), and one KO was related to siderophore production (K12241 pyochelin biosynthesis protein). Notably, the KO with the highest abundance was arthrofactin-type cyclic lipopeptide synthetase (K15659). We then retrieved the contigs assigned to the corresponding KOs. A total of 5 entries were found for hydrogen cyanide synthase, 3 entries for pyochelin biosynthesis protein, and 47 entries for arthrofactin-type cyclic lipopeptide synthetase. A further NCBI BLAST search found that all five contigs containing hydrogen cyanide synthase were assigned to *Pseudomonas*. Among the three contigs containing genes encoding the pyochelin biosynthesis protein, one contig showed high similarity with *Streptomyces*, while the other two had no significant results. As to the contigs encoding arthrofactin-type cyclic lipopeptide synthetase, 38 out of the 47 contigs belonged to *Pseudomonas*, and the rest were identified as *Streptomyces, Burkholderia,* and *Xanthomonas*. In the root endosphere, only one KO (K21003) was shown to be more abundant in GZ36, annotated as the polysaccharide biosynthesis protein PslJ.

In the phyllosphere, 226 KOs were more abundant in the leaf epiphytes of GZ36, whereas only 26 KOs showed higher abundance in HD (Supplementary material S2). Among the KOs enriched in GZ36, two KOs were related to polyketide synthase (K12434 polyketide synthase 7 and K12437 polyketide synthase) (Supplementary material S1; Supplementary Figure S3). However, in the endophytic leaf compartment, no statistical differences were observed in KOs between the two varieties.

### Positive selection and biosynthetic gene clusters in the root microbiota were associated with the disease-resistant variety

By metagenomic binning and further refinement, we obtained three high-quality metagenome-assembled genomes (MAGs) and eight high-quality MAGs from tobacco root and leaf microbiota, respectively (Supplementary material S1; Supplementary Table S1). The MAGs from the root microbiota were assigned to Rhodocyclaceae, *Pseudomonas*, and *Streptomyces*, whereas MAGs from the leaf microbiota were assigned to *Agrobacterium*, *Frateuria*, *Kosakonia*, *Neokomagataea*, *Pantoea*, *Pseudomonas*, and *Stenotrophomonas*.

To investigate the selective pressures driving protein evolution within microorganisms inhabiting the two varieties, we analyzed the polymorphism of codons in each MAG. By splitting each single codon variant (SCV) into its synonymous and non-synonymous proportions, we estimated per-site rates of synonymous polymorphism and non-synonymous polymorphism as pS and pN. The ratio between pN and pS (pN/pS) was calculated as a proxy for evolutionary pressure.

**Figure 5:**
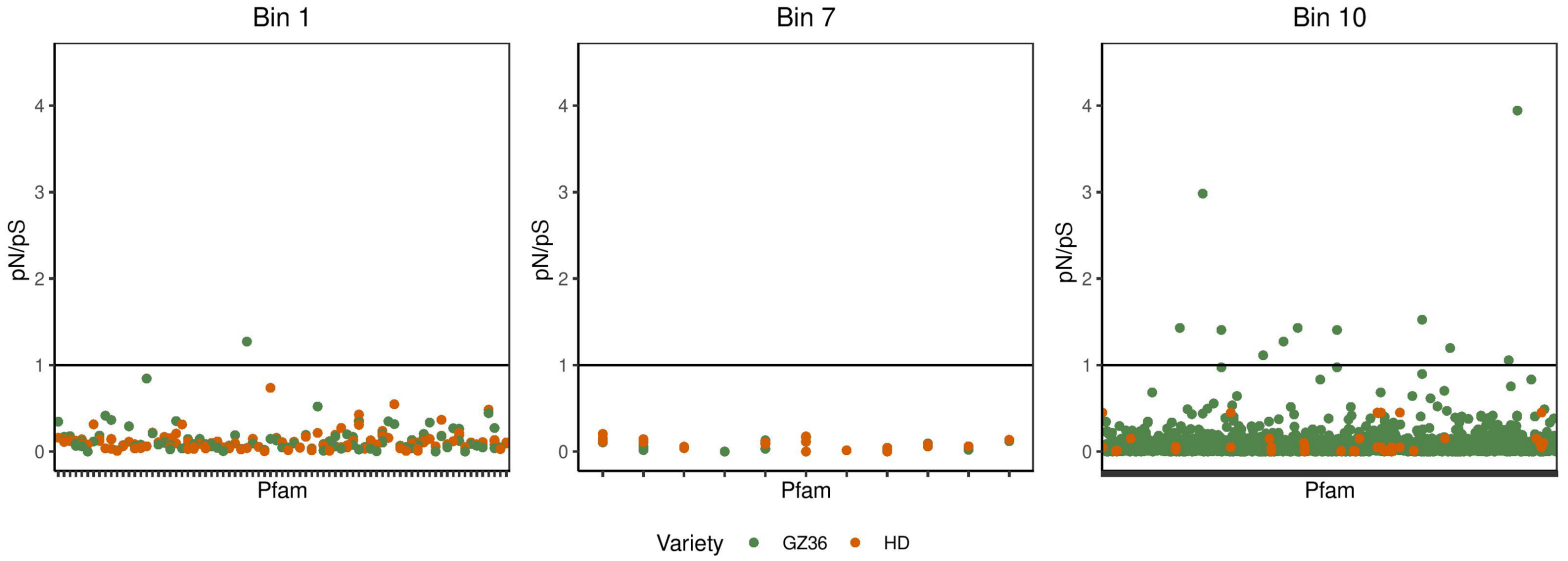
The pN/pS in the metagenome-assembled genomes retrieved from the root microbiota. The green dots represent the Pfams from the disease-resistant variety GZ36, while the red dots represent the Pfams from the disease-susceptible variety HD.

In the phyllosphere, we found the polymorphism to be overwhelmingly synonymous. None of the pN/pS exceeded 1 (Supplementary material S1; Supplementary Figure S4). In the root microbiota, the occurrence of pN/pS higher than 1 was observed in two MAGs, namely bin 1 (assigned to *Streptomyces*) and bin 10 (assigned to *Pseudomonas*) (Figure 5). A closer investigation indicated that 4 proteins in bin 10 showed a pN/pS value higher than 1 in GZ36 but not in HD, suggesting a possible positive selection of the 4 proteins in GZ36. The four proteins were annotated with functions of iron-containing redox enzyme (PF14518.9), inhibitor of vertebrate lysozyme (Ivy) (PF08816.14), carbohydrate kinase (PF01256.20), and YjeF-related protein N-terminus (PF03853.18) (Supplementary material S1; Supplementary Table S2).

We further searched the biosynthetic gene clusters (BGCs) of the MAG (bin 10) assigned to *Pseudomonas*. A total of six BGCs, including NRPS (non-ribosomal peptide synthetase), NRPS-like, RiPP-like (RiPP: ribosomally synthesized and post-translationally modified peptide product), redox-cofactor, hserlactone (homoserine lactone cluster), and aryl polyene (aryl polyene cluster), were found to be present in this MAG (Supplementary material S1; Supplementary Table S3). Notably, the compound of the NRPS BGC was predicted to be brabantamide A (with 80% gene similarity), and the compound of the hserlactone BGC was likely to be corpeptin A/corpeptin B (72% gene similarity), according to the results of the KnownClusterBlast analysis searched against the Minimum Information about a Biosynthetic Gene (MIBiG) cluster repository.

## Discussion

In this study, our comparative study revealed that the disease-resistant tobacco variety GZ36 and the disease-susceptible variety HD assembled distinct bacterial microbiota. Notably, we found that the resistant variety exhibited higher complexity of the microbial network as well as trends of higher diversity. Recent studies have revealed that microbiome network connectivity and composition are linked to disease resistance in strawberry plants (Hassani et al., 2023). Consistent with previous studies, our results suggest that higher complexity and diversity may contribute to the disease resistance of the tobacco variety GZ36.

### Potential biocontrol and growth-promoting bacteria were enriched in the rhizosphere of the resistant variety, whereas *Ralstonia* was enriched in the susceptible variety

Three genera, including *Pseudomonas*, *Ensifer*, and *Sphingopyxis*, appeared with higher abundance in the variety GZ36 based on both amplicon and metagenomic sequencing. The metagenomic analysis suggested that the enriched *Pseudomonas* belonged to species *SHC52.* The strain *Pseudomonas SHC52* was reported to be a biocontrol agent with antimicrobial activity, which could inhibit the hyphal growth of *Rhizoctonia solani* and various other fungal, oomycete, and bacterial pathogens (Schmidt et al., 2014). *Ensifer* is a genus of nitrogen-fixing bacteria, which could promote the growth and health of host plants (Rogel et al., 2001). Moreover, *Ensifer adhaerens*, as suggested by the metagenome sequencing, could attach to other bacteria and may cause the lysis of these bacteria, acting as a bacterial predator (Germida and Casida, 1983). In contrast to the known beneficial or biocontrol functions of the above two strains, *Sphingopyxis* was less studied and is most likely a commensal bacteria (Sharma et al., 2021).

The highly disease-susceptible tobacco variety, however, exhibited a higher abundance of *Ralstonia,* which could be further classified as *Ralstonia pickettii* and *Ralstonia* sp. *NFACC01*. Although the two strains were non-pathogenic (Ryan et al., 2006), they might largely overlap with the pathogen *Ralstonia solanacearum* in niches and resources (Wei et al., 2018). Thus, it is highly possible that *Ralstonia solanacearum* is more likely to survive in the rhizosphere of this variety, which further leads to disease susceptibility.

### Biocontrol-related KOs were enriched in the rhizosphere of the disease-resistant variety

In the rhizosphere, genes encoding hydrogen cyanide synthase were found to be more abundant in the variety GZ36. Hydrogen cyanide (HCN) is a well-known poison. Some HCN-producing bacteria have important effects against plant diseases (Sehrawat et al., 2022). Historically, this effect has been attributed to a direct poisonous effect (Michelsen and Stougaard, 2012). However, recent studies demonstrated that HCN plays a positive role in the plant response to pathogens independently of its toxicity, and the bacteria stimulate plant growth depending on their capacity to emit HCN (Díaz-Rueda et al., 2023).

In addition, the pyochelin biosynthesis protein showed higher abundance in the disease-resistant variety GZ36. Pyochelin is a siderophore to assimilate iron. It has been shown that siderophores serve a role beyond merely transporting iron. They are crucial mediators, facilitating interactions among microbial communities and the eukaryotic hosts they reside in (Kramer et al., 2019). Competition for iron through the secretion of siderophores has long been considered a major mechanism in the biological control of plant diseases (Miethke and Marahiel, 2007). Particularly, it has been experimentally confirmed that pyochelin-producing bacteria as well as purified pyoverdines could inhibit the growth of *Ralstonia solanacearum* (Gu et al., 2020).

Notably, arthrofactin-type cyclic lipopeptide synthetase appeared to be the most abundant ortholog in the enriched KOs. Cyclic lipodepsipeptides (CLPs) are secondary metabolites produced by non-ribosomal peptide synthetases (NRPSs) with antimicrobial functions (Raaijmakers et al., 2006). Among them, arthrofactin is one of the most potent biosurfactants (Geudens and Martins, 2018). Several CLP-producing bacteria have been inscribed for the biocontrol of plant diseases associated with CLP metabolite production (Arbeit et al., 2004).

Thus the above-mentioned KOs might be promising candidate traits potentially related to pathogen suppression. Interestingly, contigs containing corresponding genes were mostly assigned to *Pseudomonas* (and a small part to *Streptomyces*), which was consistent with the taxonomic results that *Pseudomonas* was enriched in the resistant variety.

It is suggested that the plant microbiome can determine the outcome of pathogen infections. Plants may establish interactions with protective microbes before pathogens arrive, preventing the onset of diseases (Pereira et al., 2023). While the microbiome is suggested to be linked with the disease resistance of strawberries (Hassani et al., 2023), our results provide possible explanations in terms of the functions provided by the plant microbiota. Recent studies have shown that plant genetic traits could determine the assembly of plant microbiota, which in turn prevent pathogen invasion (Escudero-Martinez et al., 2022; Oyserman et al., 2022). The plant and the associated microbiota thus form a self-reinforcing immunity and a recruitment loop, coining the concept “holobiont.” However, we acknowledge that the results presented in our study represent a plausible correlation rather than concrete causality. Further studies are still needed to corroborate the benefits of the microbiota.

### The positive selection indicated by pN/pS

In this study, four proteins were observed with pN/pS ratios >1 in GZ36 but not in HD in the MAG assigned to *Pseudomonas*, indicating that the four proteins might have been under positive selection in the resistant variety. The protein with the highest pN/pS was assigned to the function of an iron-containing redox enzyme. Iron is not only an essential nutrient for the growth of microorganisms but is also critically involved in the redox response (Cornelis et al., 2011). Competition for iron was shown to drive phytopathogen control in the natural rhizosphere (Gu et al., 2020). The second protein, an inhibitor of vertebrate lysozyme (Ivy), is reported as a stress response to cell wall damage (Abergel et al., 2007). It is possible that the two proteins of *Pseudomonas* were under positive selection.

### NRPS identified in the MAG

The search of BGCs in the MAG assigned to *Pseudomonas* suggested the presence of NRPS, which was likely to be brabantamide A. Brabantamide A has been isolated from *Pseudomonas fluorescens* DSM 11579 (Thirkettle et al., 2000) and *Pseudomonas brassicacearum* (Andersson et al., 2012). The genetic origin and biosynthetic pathways of brabantamide A were elucidated clearly in *Pseudomonas* sp., *SHC52* (Schmidt et al., 2014). Significant antibacterial and antifungal activity was reported with brabantamide A. This is also consistent with the taxonomic results showing that *Pseudomonas* sp., *SHC52* was enriched in the resistant variety. With the high similarity of brabantamide A in our MAG, it is highly possible that the *Pseudomonas* obtained by metagenome binning is a promising biocontrol agent.

Additional investigations are warranted to isolate the bacterial strain *Pseudomonas* and substantiate its protective efficacy against plant pathogens. Subsequent research endeavors should focus on elucidating the mechanisms underlying this protective function, potentially through genomic and metabolomic analyses. Moreover, experimentation aimed at determining the transferability and reproducibility of the protective effect through microbial transplantation would provide valuable insights. These efforts could pave the way for innovative applications in agriculture, such as developing biocontrol agents or engineering plant microbiomes for enhanced disease resistance.

## Conclusion

In this study, we carried out a comparative microbiome study of two tobacco varieties, which are highly resistant and susceptible to the common tobacco diseases, respectively. Our results revealed that the resistant variety assembled microbiota with higher network complexity and diversity. Notably, *Pseudomonas SHC52,* a known biocontrol agent, was enriched in the rhizosphere of the resistant variety, whereas *Ralstonia*, a genus containing the known causative agent, was enriched in the susceptible variety. Moreover, biocontrol functions such as hydrogen cyanide synthase, pyochelin biosynthesis, and arthrofactin-type cyclic lipopeptide synthetase were enriched in the resistant variety, which were largely derived from *Pseudomonas*, as indicated by sequence retrieval and BLAST analysis. Metagenome-assembled genomes were further obtained by binning. In the assembled genome assigned to *Pseudomonas*, positive selection was suggested for the resistant variety. In addition, a non-ribosomal peptide synthetase encoding brabantamide A was identified, which was known to have antimicrobial activity. Collectively, our study suggests *Pseudomonas* as a promising biocontrol agent that might contribute to the disease resistance of the host plant.

## Data availability statement

The sequencing datasets presented in this study can be found in GSA database (https://bigd.big.ac.cn/gsa/) under the accession number CRA008960.

## Author contributions

LY: Data curation, Formal analysis, Visualization, Writing – original draft. YG: Data curation, Investigation, Methodology, Writing – review & editing. HY: Conceptualization, Data curation, Writing – review & editing. SL: Writing – review & editing. YZ: Writing – review & editing. CG: Writing – review & editing. TW: Writing – review & editing. LH: Conceptualization, Funding acquisition, Project administration, Supervision, Writing – review & editing.

## Footnotes

^1^ http://huttenhower.sph.harvard.edu/kneaddata

^2^ https://github.com/wwood/CoverM
