# Supplementary material for: Distinct microbiota assembly and functional patterns in disease-resistant and susceptible varieties of tobacco
Source: Front Microbiol. 2024 Mar 1;15:1361883. doi: 10.3389/fmicb.2024.1361883 (PMC10940526; doi:10.3389/fmicb.2024.1361883)
Supplement: Supplementary file 1 [file Data_Sheet_1.zip › Supplementary material S1.docx]

Supplementary figures

Fig. S1 The keystone OTUs identified in the Zi-Pi plots of each molecular ecological network

Fig. S2 The taxonomic differences between the two varieties based on metagenomic sequencing using Linear discriminant analysis Effect Size (LEfSe)

Fig. S3 The KOs differed in abundance in the epiphytic phyllosphere between the two varieties

Fig. S4 Polymorphism distribution patterns in all the bins assembled from the tobacco phyllosphere

Supplementary tables

Table S1 The completeness, contamination, coverage, and phylogeny of the metagenome assembled genomes (MAGs) reconstructed from the tobacco metagenome in (a) root and (b) leaf compartments.

Table S2 The pN/pS differed between the two varieties in tobacco metagenome (values>1)

Table S3 The biosynthetic gene clusters in bin 10 in tobacco root microbiome predicted by antiSMASH.

Fig. S1 The keystone OTUs identified in the Zi-Pi plots of each molecular ecological network


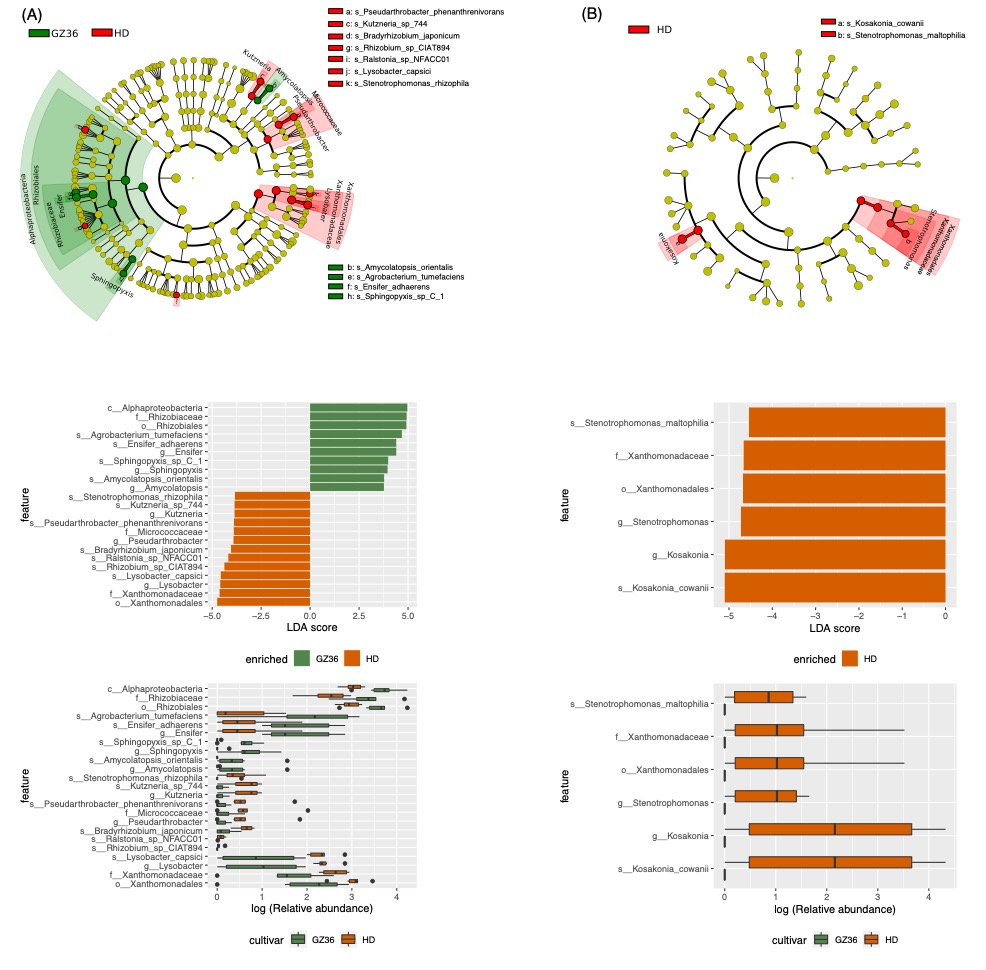


Fig. S2 The taxonomic differences between the two varieties based on metagenomic sequencing using Linear discriminant analysis Effect Size (LEfSe). (A) The rhizosphere and root endosphere were combined as root samples. (B) The epiphytic and endophytic leaf compartments were combined as phyllosphere.

Fig. S3 The KOs differed in abundance in the epiphytic phyllosphere between the two varieties.

Fig. S4. Polymorphism distribution patterns in all the bins assembled from the tobacco phyllosphere. Each dot represent a pN/pS for a protein.

Supplementary tables

Table S1 The completeness, contamination, coverage, and phylogeny of the metagenome assembled genomes (MAGs) reconstructed from the tobacco metagenome in (a) root and (b) leaf compartments.

(a) Metagenome assembled genomes reconstructed from the tobacco root metagenome

| Bin ID | Comple-  teness | Contami-  nation | GC | N50 | Size | Taxonomy |
| --- | --- | --- | --- | --- | --- | --- |
| bin.1 | 80.29 | 3.501 | 0.707 | 7315 | 6014143 | *Streptomyces acidiscabies* |
| bin.7 | 90.31 | 2.618 | 0.607 | 7441 | 2650578 | Rhodocyclaceae |
| bin.10 | 86.33 | 3.577 | 0.612 | 13139 | 4710232 | *Pseudomonas_E sp000801235* |

(b) Metagenome assembled genomes reconstructed from the tobacco leaf metagenome

| Bin ID | Comple-teness | Contami-  nation | GC | N50 | Size | Taxonomy |
| --- | --- | --- | --- | --- | --- | --- |
| bin.1 | 99.19 | 1.762 | 0.592 | 68500 | 5637370 | *Agrobacterium sp900013535* |
| bin.3 | 94.98 | 3.403 | 0.663 | 22488 | 4476285 | *Stenotrophomonas lactitubi* |
| bin.4 | 91.93 | 0.799 | 0.610 | 20779 | 5424294 | *Pseudomonas_E* |
| bin.5 | 90.21 | 1.043 | 0.545 | 8007 | 1979734 | *Neokomagataea* |
| bin.6 | 97.60 | 0.909 | 0.575 | 74793 | 2824943 | *Frateuria* |
| bin.7 | 89.66 | 3.135 | 0.636 | 17646 | 5978141 | *Pseudomonas_E protegens* |
| bin.8 | 93.80 | 3.406 | 0.566 | 9065 | 3942290 | *Kosakonia cowanii* |
| bin.9 | 87.62 | 2.855 | 0.544 | 7586 | 3700174 | *Pantoea ananatis* |

Table S2 The pN/pS differed between the two varieties in tobacco metagenome (values>1)

| Pfam | Mean value of pN/pS | |
| --- | --- | --- |
|  | GZ36 | HD |
| PF14518.9: Iron-containing redox enzyme | 1.98 | NA |
| PF08816.14: Inhibitor of vertebrate lysozyme (Ivy) | 1.21 | NA |
| PF01256.20: Carbohydrate kinase | 1.19 | NA |
| PF03853.18: YjeF-related protein N-terminus | 1.19 | NA |

Table S3 The biosynthetic gene clusters in bin 10 in tobacco root microbiome predicted by antiSMASH.

| Region | Type | Most similar known cluster | Similarity |
| --- | --- | --- | --- |
| Region 31.1 | hserlactone | corpeptin A/corpeptin B | 72% |
| Region102.1 | arylpolyene | APE Vf | 30% |
| Region 171.1 | NRPS-like | - | - |
| Region 284.1 | NRPS | brabantamide A | 80% |
| Region 339.1 | redox-cofactor | lankacidin C | 13% |
| Region 354.1 | RiPP-like | - | - |
